# Epidemiology of trauma admissions in a level 1 trauma center in Northern Italy: a nine-year study

**DOI:** 10.1007/s13304-021-00991-y

**Published:** 2021-05-18

**Authors:** Margherita Difino, Roberto Bini, Elisa Reitano, Roberto Faccincani, Fabrizio Sammartano, Laura Briani, Stefania Cimbanassi, Osvaldo Chiara

**Affiliations:** 1 Vita-Salute San Raffaele University, Via Olgettina 60, 20132 Milan, Italy; 2 General Surgery and Trauma Team, ASST Niguarda, Milano, Piazza Ospedale Maggiore 3, 20162 Milan, Italy; 3 University of Milano, Festa del Perdono 7, 20122 Milan, Italy; 4 Emergency Department, IRCCS San Raffaele, Via Olgettina, 60, 20132 Milan, Italy

**Keywords:** Trauma center, Urban area, Epidemiology of trauma, Emergency department, Overtriage

## Abstract

The aim of this study was to analyze the results of 9 years of trauma care and data collection in a level 1 urban trauma center in Northern Italy. Overall, 6065 patients have been included in the study; the number of patients managed yearly has doubled between 2011 and 2019. This rise mostly involved patients with injury severity score (ISS) < 16. Most injuries (94%) were blunt. Road traffic accidents, especially involving motorcycles, were the most common cause of injury. Self-inflicted injuries were responsible for less than 5% of trauma but they were severe in 56% of cases. The median age was 38 and it remained constant over the years; 43% of patients had 14–39 years of age. Different characteristics and patterns of injury were observed for each age group and gender. Males were more likely to be injured in the central years of life while females presented a trimodal pattern in the age distribution. Young adults (14–39 years old) were overall at higher risk of self-harm. Overall mortality was equal to 5.2%. Most deceased were male and ≥ 65 years of age.

## Introduction

Trauma is the 3rd cause of death in western countries, after cardiovascular diseases and tumors, and the first cause in population with less than 40 years of age [1], constituting a relevant social and economic burden.

Registries collecting data regarding injured patients are used to improve knowledge on trauma and its patterns, with the objective to document in-hospital trauma care in the acute setting [2]. Such registries have multiple applications, e.g. the generation of hypothesis, the planning of protocols and studies and the monitoring of the effectiveness of new interventions; on an administrative level, registries are essential for planning resource allocation by estimating needs in materials and human resources, and are used for awareness campaigns as well as occupational surveillance [2].

One of the limitations of studies conducted with data collected in trauma registries is that registries are not always homogeneous and, therefore, the outcomes of the analyses cannot be compared with acceptable validity, nor can they be incorporated into large-scale studies of epidemiological significance [3].

Niguarda Hospital, located in Milan, Italy, is the level 1 Trauma Center (TC) of an urban area of over 3 million inhabitants [4], recognized as a highly specialized TC in the regional integrated trauma system (SIAT). The Trauma Team (TT) of Niguarda Hospital has been keeping a trauma registry, containing all the relevant data on trauma patients managed by the TT, since October 2002.

The aim of this study is to investigate the evolution of the demographics of trauma patients and patterns of trauma injuries over a timeframe that ranges from January 1, 2011 to December 15, 2019. The results of almost a decade of trauma care and data collection in an Italian level 1 trauma center are reported. To the best of our knowledge, this constitutes the largest series of injured patients admitted at a single institution in Italy entailing the activation of the hospital TT.

## Materials and methods

The TT has been collecting data prospectively since 2002. The registry is kept constantly updated by the emergency surgeons of the TT and it is periodically revised by the head of the department.

At the beginning data were recorded in a FileMaker version 6 which was changed in 2011 into a Microsoft ACCESS^®^ (version 16.0.10827.20118) database to simplify elaboration. The institution of the trauma registry for all major trauma admitted to our TC has been approved by the Niguarda Ethical Committee Milano Area 3 (record number 534–102,018).

All victims of injuries, both blunt and penetrating, admitted to Niguarda Hospital with the activation of TT by the pre-hospital emergency service or, in a small proportion, following in-hospital triage, were included in the registry. Patients reporting exclusively burns, who are managed by the specialized Burn Unit in Niguarda Hospital, were excluded.

The data used for the present study include the patients’ demographic information, date of the incident and time spent on scene, type and time of transport, presence of advanced life support team on scene, vital parameters (heart rate, respiratory rate, systolic blood pressure, Glasgow Coma Scale) both in pre-hospital setting and upon arrival in the emergency department, mechanism of injury (MOI), information on the emergency procedures performed (both pre- and in-hospital) damage control procedures, non-urgent surgery, definitive balance of injuries with the relative abbreviated injury score (AIS) per body district (version 2008), injury severity score (ISS). The case fatality ratio (CFR) of each MOI was calculated.

The MOIs were classified as motorcycle, motor vehicle, pedestrian, bicycle, falls from heights, crush injury, stab wound, gunshot wound (GSW), or other. ‘Other’ includes also trauma deriving from explosions, battery, interaction with animals (bites, general aggression by big mammals—e.g. cows), sport injuries, undefined traumas due to impact with blunt objects, and trauma as a result of railway and airplane crashes.

For the purpose of analysis and reporting, a further stratification of the patients was deemed necessary. Similar to the classification of intent by Ringdal et al. [5] in their revision of the Utstein template, injuries were stratified based on the “role of human intent in the occurrence of an injury” [5] as intentional (either self-inflicted or resulting from assault) or un-intentional (road traffic, work-related injuries, accidental falls and other accidental injuries).

The population was stratified by age in five groups: pediatric age (0–13), young adults (14–39), adults (40–64), and the patients with more than 65 years. Older victims of trauma were further subdivided in two groups [6]: elder adults (65–75) and elderly (> 75).

Patients with ISS ≥ 16 were considered severely injured. Overtriage was defined as the rate of patients admitted to a level 1 TC with TT activation and an ISS < 16, as suggested by the American College of Surgeons Committee on Trauma.

Those patients whose relevant data, mainly demographic, were missing and could not be retrieved from the medical records were excluded from the study.

Given the retrospective nature of the study, a specific ethical review board approval was not required.

Data were recorded in a computerized spreadsheet (Microsoft Excel 2016, Microsoft Corporation, Redmond, WA) and analyzed with statistical software (IBM Corp. Released 2012, IBM SPSS Statistics for Windows, version 21.0, Armonk, NY, IBM Corp.). Data visualization was obtained with R coding. The sample distribution was evaluated with Kolmogorov–Smirnov and Shapiro–Wilk tests, resulting in a non-Gaussian distribution for any of the examined variables. Continuous data were compared by independent sample Kruskar–Wallis test, and categorical data using Pearson’s chi-square test. *P* values below 0.05 were considered statistically significant. Logistic regression analysis was performed to identify independent predictors of mortality.

## Results

**Fig. 1:**
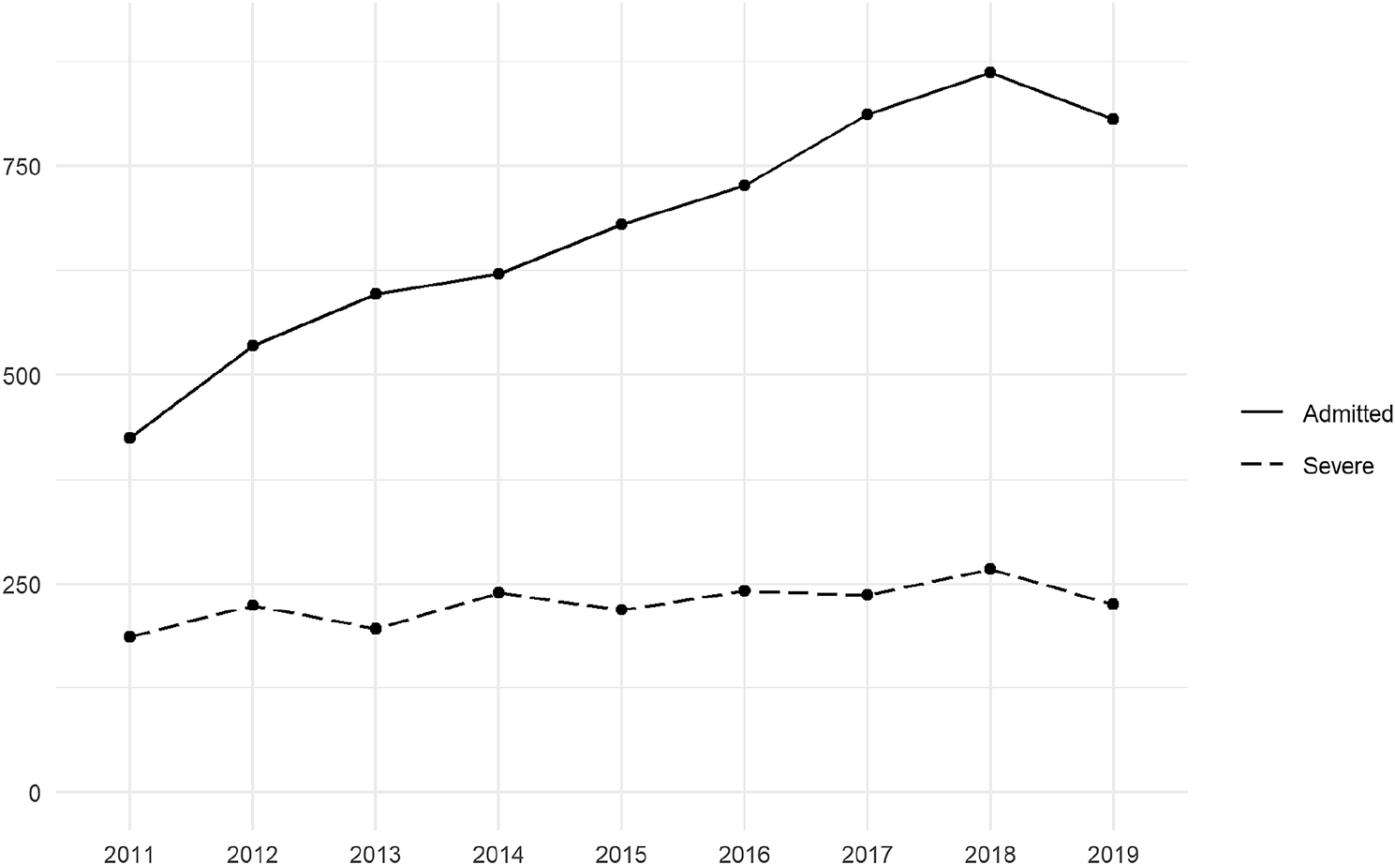
Admission to Niguarda ED (2011–2019)

During the 9-year period covered by this study, 6065 trauma patients were managed by the TT in Niguarda Hospital. The number of patients assisted yearly almost doubled between 2011 and 2019, increasing from 425 to 806 per year, as shown in Fig. 1.

### ISS

The median ISS of the overall population was 9 (IQR 1–20). The severely injured (ISS ≥ 16) were 2038 (33.6% of the total), among whom the median ISS was 25.50 (IQR 20–36).

Even though a mild rise in the total number of severely injured was observed in correspondence to the increase in patients over the 9 years (Fig. 1), the ratio of patients with ISS ≥ 16 has shown a reduction from 44 to 28%, suggesting an increase in overtriage from 56 to 72%.

### Etiology and mechanism

Blunt injuries prevailed over penetrating ones and represented 94% of the total, oscillating over the years between 92 and 97%. Results regarding MOI are displayed in Table 1. Motorcycle collisions constituted 26% of the causes of injury followed by falls and motor vehicle accidents. Except for some oscillations, the ratios of the MOIs remained approximately constant over the years.

**Table 1:** Mechanisms of injury over the years

	Years									
	2011 *n* (%)	2012 *n* (%)	2013 *n* (%)	2014 *n* (%)	2015 *n* (%)	2016 *n* (%)	2017 *n* (%)	2018 *n *(%)	2019 *n* (%)	Total per mechanism *n* (%)
Motorcycle	121 (28.5)	171 (32.0)	142 (23.8)	157 (25.3)	185 (27.2)	183 (25.2)	192 (23.6)	218 (25.3)	212 (26.3)	1581 (26.1)
Motor vehicle	75 (17.6)	96 (17.9)	111 (18.6)	105 (16.9)	128 (18.8)	151 (20.8)	187 (23.0)	188 (21.8)	161 (20.0)	1202 (19.8)
Fall	86 (20.2)	88 (16.4)	113 (18.9)	143 (23.0)	140 (20.6)	157 (21.6)	140 (17.2)	168 (19.5)	152 (18.9)	1187 (19.6)
Pedestrian	62 (14.6)	75 (14.0)	98 (16.4)	112 (18.0)	104 (15.3)	127 (17.5)	140 (17.2)	151 (17.5)	119 (14.8)	988 (16.3)
Bicycle	26 (6.1)	41 (7.7)	39 (6.5)	46 (7.4)	40 (5.9)	56 (7.7)	74 (9.1)	67 (7.8)	57 (7.1)	446 (7.4)
Stab wound	22 (5.2)	29 (5.4)	32 (5.4)	29 (4.7)	35 (5.1)	21 (2.9)	39 (4.8)	24 (2.8)	41 (5.1)	272 (4.5)
Other	18 (4.2)	20 (3.7)	36 (6.0)	19 (3.1)	21 (3.1)	16 (2.2)	27 (3.3)	27 (3.1)	39 (4.8)	223 (3.7)
Crush injury	9 (2.1)	13 (2.4)	16 (2.7)	8 (1.3)	12 (1.8)	13 (1.8)	9 (1.1)	14 (1.6)	14 (1.7)	108 (1.8)
Gunshot wound	6 (1.4)	2 (0.4)	10 (1.7)	1 (0.2)	15 (2.2)	3 (0.4)	3 (0.4)	4 (0.5)	7 (0.9)	51 (0.8)
Unknown	0 (0)	0 (0)	0 (0)	1 (0.2)	0 (0)	0 (0)	1 (0.1)	1 (0.1)	4 (0.5)	7 (0.1)
Total per year	425	535	597	621	680	727	812	862	806	6065

### Age

**Fig. 2:**
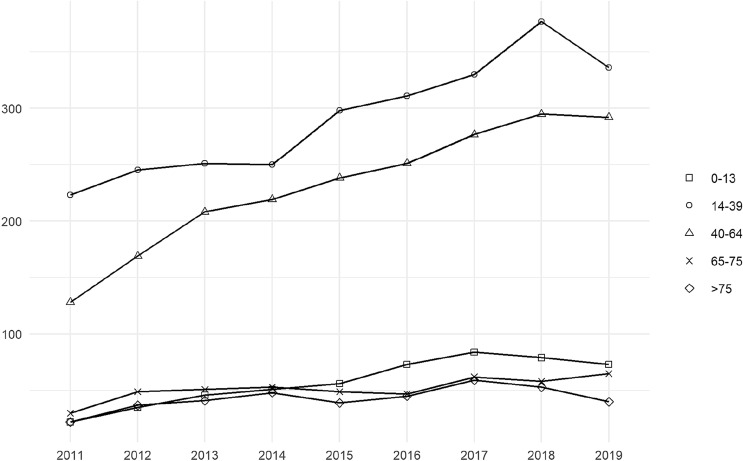
Age distribution (2011–2019)

The median age of the overall population was 38 (IQR 25–53) years, which remained constant over the years with only minimal variations. Most patients (*n* = 2621, 43%) were young adults (14–39 years old), followed by adults (40–64 years old), which represented 34% of the sample (*n* = 2077). Children were 519 (9%), whereas the elder patients, further subdivided in two groups (65–75 and > 75) were 464 (8%) and 384 (6%), respectively. These proportions remained almost constant throughout the period of the study (Fig. 2).

With respect to the intentionality of the trauma (Table 2), un-intentional injuries were more common than intentional ones, in particular, road traffic accidents were responsible for 69.5% of all injuries, followed by accidental falls (12.3%).

**Table 2:** Internationality of trauma over the years

	Years									
	2011 *n* (%)	2012 *n* (%)	2013 *n* (%)	2014 *n* (%)	2015 *n* (%)	2016 *n* (%)	2017 *n* (%)	2018 *n* (%)	2019 *n* (%)	Total *n* (%)
Un-intentional										
Road traffic	284 (77.2)	383 (81.3)	389 (76.3)	419 (75.9)	460 (77.2)	516 (78.4)	592 (80.9)	624 (79.4)	549 (76.9)	4216 (78.3)
Work related	29 (7.9)	22 (4.7)	31 (6.1)	27 (4.9)	31 (5.2)	35 (5.3)	35 (4.8)	36 (4.6)	49 (6.9)	295 (5.5)
Accidental falls	46 (12.5)	52 (11.0)	69 (13.5)	93 (16.8)	93 (15.6)	99 (15.0)	89 (12.2)	106 (13.5)	101 (14.1)	748 (13.9)
Other accidental	9 (2.4)	14 (3.0)	21 (4.1)	13 (2.4)	12 (2.0)	8 (1.2)	16 (2.2)	20 (2.5)	15 (2.1)	128 (2.4)
Total un-intentional	368	471	510	552	596	658	732	786	714	5387
Intentional										
Falls or self-inflicted wounds	23 (46.9)	24 (42.1)	29 (39.2)	26 (44.8)	37 (46.3)	31 (55.4)	28 (38.9)	43 (63.2)	36 (45.6)	277 (46.7)
Assault by others	26 (53.1)	33 (57.9)	45 (60.8)	32 (55.2)	43 (53.8)	25 (44.6)	44 (61.1)	25 (36.8)	43 (54.4)	316 (53.3)
Total intentional	49	57	74	58	80	56	72	68	79	593
Unknown intent	8	7	13	11	4	13	8	8	13	85

**Fig. 3:**
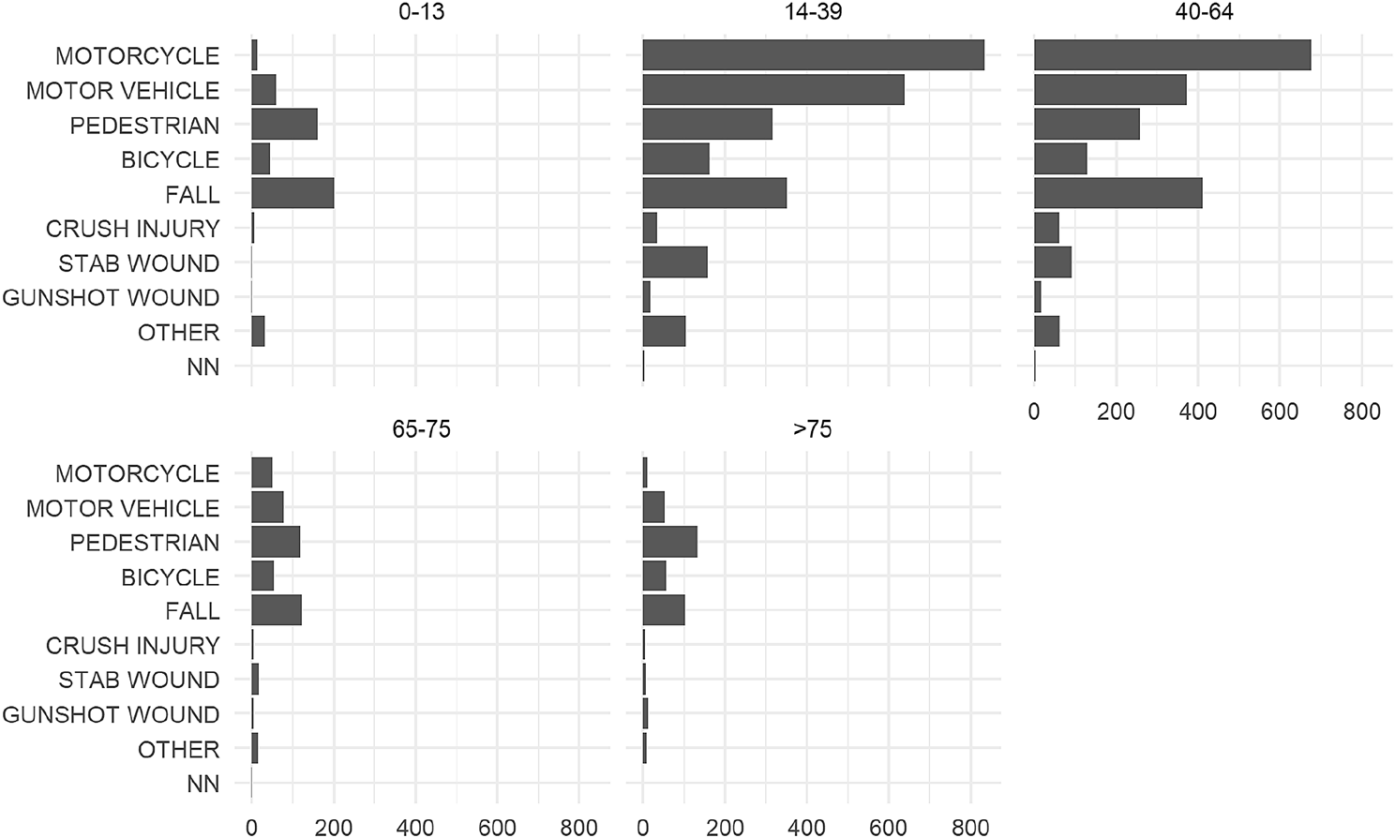
Mechanisms of injury per age group

In pediatric age, the most common causes of trauma were falls (39%) and pedestrians struck by vehicles (31%). Young adults and adults were more commonly involved in motorcycle (32%) and motor vehicle (24%) collisions, while falls were proportionally less common, making up for only 13% of injuries. Among the elderly, falls and injured pedestrians predominated, representing, respectively, 27% and 35% of all the injuries in this age group (Fig. 3). A dynamic evaluation of the trends of the MOIs and their intentionality for each age group over the 9 years showed an increase in accidental fall, from 9 to 28% among patients with more than 75 years of age and a decrease from 27 to 21% in patients with an age between 65 and 75 years.

Focusing on each age group and gender (Table 3), the ratio of severely injured increased steadily with age, from 16% in pediatric age up to 46% and 57% among elder adults and elderly, respectively. Interestingly, among intentional trauma in young adults and adults, self-inflicted lesions were more likely to be severe than injuries deriving from assault, with the former having an ISS ≥ 16 in 56% of cases and the latter in 19% of cases.

**Table 3:** Distribution of severe injuries by age group and gender

*N*	Age groups										Total
	0–13		14–39		40–4		65–75		> 75		
	519		2621		2077		464		384		6065
ISS ≥ 16 [*n* (%)]	83 (16.0)		747 (28.5)		773 (37.2)		214 (46.1)		221 (57.6)		2038 (33.6)
	ISS ≥ 16										
	Male *n* (%)	Female *n* (%)	Male *n* (%)	Female *n* (%)	Male *n* (%)	Female *n* (%)	Male *n* (%)	Female *n* (%)	Male *n* (%)	Female *n* (%)	Total *n* (%)
Mechanism											
Motorcycle	1 (2.1)	–	246 (43.2)	32 (18.1)	241 (39.4)	20 (12.3)	17 (11.3)	–	4 (3.2)	–	561 (27.5)
Motor vehicle	4 (8.5)	4 (11.1)	92 (16.1)	55 (31.1)	76 (12.4)	27 (16.7)	15 (9.9)	10 (15.9)	18 (14.3)	4 (4.2)	305 (15.0)
Pedestrian	11 (23.4)	9 (25.0)	53 (9.3)	30 (16.9)	55 (9.0)	54 (33.3)	34 (22.5)	29 (46.0)	35 (27.8)	48 (50.5)	358 (17.6)
Bicycle	6 (12.8)	4 (11.1)	30 (5.3)	11 (6.2)	41 (6.7)	11 (6.8)	22 (14.6)	6 (9.5)	24 (19.0)	7 (7.4)	162 (7.9)
Fall	21 (44.7)	16 (44.4)	93 (16.3)	46 (26.0)	148 (24.2)	46 (28.4)	55 (36.4)	17 (27.0)	30 (23.8)	31 (32.6)	503 (24.7)
Stab wound	–	–	23 (4.0)	1 (0.6)	9 (1.5)	2 (1.2)	1 (0.7)	–	1 (0.8)	1 (1.1)	38 (1.9)
Gunshot wound	–	–	3 (0.5)	–	7 (1.1)	–	1 (0.7)	–	9 (7.1)	–	20 (1.0)
Crush injury	1 (2.1)	–	12 (2.1)	1 (0.6)	20 (3.3)	–	2 (1.3)	–	1 (0.8)	1 (1.1)	38 (1.9)
Other	3 (6.4)	3 (8.3)	18 (3.2)	1 (0.6)	14 (2.3)	2 (1.2)	3 (2.0)	1 (1.6)	4 (3.2)	3 (3.2)	52 (2.6)
Unknown	–	–	–	–	–	–	1 (0.7)	–	–	–	1 (0.0)
Intentionality											
Un-intentional											
Road traffic	22 (46.8)	17 (47.2)	420 (73.7)	128 (72.3)	415 (67.9)	112 (69.1)	88 (58.3)	45 (71.4)	81 (64.3)	59 (62.1)	1387 (68.1)
Work related	–	–	29 (5.1)	–	63 (10.3)	1 (0.6)	5 (3.3)	1 (1.6)	1 (0.8)	–	100 (4.9)
Accidental falls	18 (38.3)	13 (36.1)	25 (4.4)	16 (9.0)	77 (12.6)	20 (12.3)	45 (29.8)	10 (15.9)	16 (12.7)	20 (21.1)	260 (12.8)
Other accidental	3 (6.4)	3 (8.3)	5 (0.9)	2 (1.1)	5 (0.8)	1 (0.6)	1 (0.7)	–	2 (1.6)	2 (2.1)	24 (1.2)
Intentional											
Falls or self-inflicted wounds	1 (2.1)	2 (5.6)	46 (8.1)	26 (14.7)	23 (3.8)	23 (14.2)	8 (5.3)	6 (9.5)	14 (11.1)	7 (7.4)	156 (7.7)
Assault by others	–	–	33 (5.8)	–	17 (2.8)	2 (1.2)	1 (0.7)	–	4 (3.2)	3 (3.2)	60 (2.9)
Unknown intent	3 (6.4)	1 (2.8)	12 (2.1)	5 (2.8)	11 (1.8)	3 (1.9)	3 (2.0)	1 (1.6)	8 (6.3)	4 (4.2)	51 (2.5)

*ISS* injury severity score

### Gender

Among the patients included in the study, 4473 were male (74%) and 1592 were female (26%). The MOIs varied with genders: among males, the most common MOI was motorcycle collisions (*n* = 1406, 31%), followed by falls (*n* = 838, 19%) and motor vehicle collisions (*n* = 775, 17%); females, were more commonly involved in pedestrian accidents (*n* = 459, 29%), motor vehicle collisions (*n* = 427, 27%) and falls (*n* = 349, 22%). Road traffic collisions were by far the most common cause of injuries for both males and females (69% and 73%, respectively), followed among males by accidental falls (*n* = 519, 12%), assault (7%) and work-related injuries (6%). Among females, road traffic accidents were followed by accidental falls (14%) and self-inflicted (7%) injuries.

**Fig. 4:**
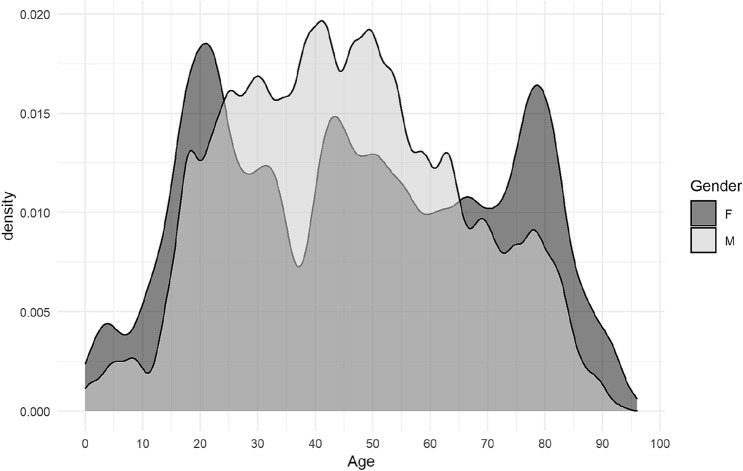
Distribution of severely injured (ISS ≥ 16) by age and gender

In the analysis of the distribution of trauma in relation to age and gender, male patients tended to be clustered in an age group between approximately between 20 and 60 years. The age distribution of females showed three peaks around approximately 20, 45, and 80 years, defining a trimodal distribution. This pattern was enhanced when focusing on the severely injured (Fig. 4).

### Intentionality

The intentionality of trauma was specific for each age group and gender, especially among the severely injured patients (Table 3). In children, almost all severe injuries were unintentional, while intentional injuries increased in the other age groups.

Among the female severely injured, self-inflicted injuries became the second cause of trauma for young women with 14–39 years of age (14.7%) and women with age comprised between 40 and 65 years old (14.2%). Accidental falls were increasingly responsible for severe injuries as female patients grow older, up to 21% of trauma in women > 75 years.

Among the male severely injured, self-inflicted injuries were the second cause of trauma for young adults (14–39 years old) and became relevant again after 75 years of age. For adults (40–64 years old) and elder adults (65–75 years old), the most common cause of trauma after road traffic accidents was accidental fall. Injuries following assault were more commonly found in young men, while work-related traumas were typical of male adults.

### Mortality

Of the 6065 patients of the trauma registry that were included in the study, 316 died; the overall mortality was equal to 5.2%, specifically 5.1% for blunt trauma and 6.8% for penetrating trauma. Most of the deceased were male (*n* = 229, 72.5%) with a median age of 59 (IQR 40–78).

Most of the deceased were 65 years old or older: 47 (15%) with an age between 65 and 75 and 94 (30%) > 75 years old. Adults (40–64 years old) who died were 101 (32%), while young adults (14–39 years old) were 64 (20%) and children represented the smallest part of the deceased population (*n* = 10, 3%). Blunt trauma constitutes 92% (*n* = 291) of lethal injuries.

The highest CFR (Table 4) was observed in GSW (25.5%), followed by falls (9.1%) and bicycle-related injuries (7.4). Almost 13% of patients with self-inflicted injuries succumbed to their injuries. Un-intentional injuries and those caused by assault were less frequently associated with the death of the patient (respectively, 3.5–4.5%). In univariate analysis, mechanism of trauma, age range and ISS ≥ 16 were found to be significantly associated with mortality, while gender was excluded from the multivariate analysis. All variables entered in the multiple regression model were confirmed independent predictors of death (Table 5).

**Table 4:** Case fatality ratio

	Deceased (*n*)	Total (*n*)	CFR (%)
Mechanism of injury			
Falls	108	1187	9.1
Pedestrian	58	988	5.9
Motorcycle	44	1581	2.8
Motor vehicle	38	1202	3.2
Bicycle	33	446	7.4
Gunshot wound	13	51	25.5
Stab wound	8	272	2.9
Crush injury	2	108	1.9
Other	11	223	4.9
Unknown	1	7	
Intentionality			
Road traffic	174	4216	4.1
Work related	13	295	4.4
Accidental fall	45	748	6.0
Other accidental injuries	–	128	0.0
Intentional falls or self-inflicted wounds	35	277	12.6
Assault by others	11	316	3.5
Unknown	38	85

**Table 5:** Logistic regression for the identification of independent predictors of death

	Univariate analysis			Multivariate analysis	
	Survived	Dead	*P* value	OR (95% CI)	*P* value
Mechanism					
Motorcycle [*n* (%)]	1537 (25.3)	44 (0.7)	≤ 0.001*	0.233 (0.047–1.151)	≤ 0.001*
Motor vehicle [*n* (%)]	1164 (19.2)	38 (0.6)			
Pedestrian [*n* (%)]	930 (15.3)	58 (1)			
Bicycle [*n* (%)]	413 (6.8)	33 (0.5)			
Fall [*n* (%)]	1079 (17.8)	108 (1.8)			
Stab wound [*n* (%)]	264 (4.4)	8 (0.1)			
Gunshot wound [*n* (%)]	38 (0.6)	13 (0.2)			
Crush injury [*n* (%)]	106 (1.7)	2 (0.1)			
Other [*n* (%)]	212 (3.5)	11 (0.2)			
Unknown [*n* (%)]	6 (0.1)	1 (0)			
Age range					
0–13 [*n* (%)]	509 (8.4)	10 (0.2)	≤ 0.001*	0.387 (0.253–0.593)	≤ 0.001*
14–39 [*n* (%)]	2557 (42.2)	64 (1.1)			
40–64 [*n* (%)]	1976 (32.6)	101 (1.7)			
65–75 [*n* (%)]	417 (6.9)	47 (0.8)			
> 75 [n (%)]	290 (4.8)	94 (1.5)			
Gender					
Male [*n* (%)]	4244 (70.0)	229 (3.8)	0.595		
Female [*n* (%)]	1505 (24.8)	87 (1.4)			
ISS ≥ 16 [*n* (%)]	1731 (28.5)	307 (5.1)	≤ 0.001*	0.014 (0.007–0.028)	≤ 0.001*

## Discussion

This study presents a retrospective review of the biggest trauma registry in Italy, where all data of trauma patients admitted with the activation of the hospital TT are included. The aim was to analyze over a 9-year period the epidemiology of patients transported by the pre-hospital system to the referral hospital of a large urban area for suspected severe trauma. Since the foundation of the TT, Niguarda was the only TC in Italy with a surgical leadership for the organization, management, inter-specialty coordination, and quality assessment of trauma care, keeping a trauma registry and university teaching of trauma and acute care surgery to medical students, nurses, general surgery residents and post-specialty master students [7]. After the institution of the regional trauma system, Niguarda Hospital has taken up the role of highly specialized level 1 TC, capable of providing definitive care to trauma patients. Italian literature on trauma is limited to the brief Italian experience [26] or focused on specific trauma samples [27, 28]. To our knowledge, this is the largest epidemiological Italian study on the topic, providing a complete snapshot of demographics and injury distribution of trauma patients in Italy. The effect of a dedicated trauma system on patients’ outcome has already been highlighted in several studies [29, 30].

This study demonstrates that the centralization of trauma patients to the level 1 TC has increased considerably over the years, but this trend was not followed by a proportional increase in admission of severely injured patients, defined as ISS ≥ 16, with a consequent marked increase in overtriage. The explanation behind this discrepancy is multifactorial. Patients with normal vital signs who have endured trauma with high-energy mechanisms, suggesting the possibility of severe injuries, are indicated to be transported to the local TC. This triage method is generally associated with a high rate of overtriage [8]. The development and implementation of many devices able to reduce the energy transmitted to the body following an impact (e.g. seatbelts, helmets, airbags in car interiors, jacket with airbags for bikers) can effectively limit the severity of injuries. The association highlighted in historical studies between some mechanisms of injury with severe trauma should be reconsidered because of low specificity.

The Italian Ministry of Health is changing pre-hospital triage rules to reduce overtriage in TC hospitals: (a) trauma patients with high-energy mechanism and stable vital signs will be admitted preferentially to level 2 TCs; (b) patients with hemodynamic instability will be always centralized to level 1 or level 2 TCs with damage control expertise, promoting the use of pre-hospital techniques of temporary hemorrhage control and blood pressure improvement (tourniquet, pelvic binder, REBOA, transfusions, vasopressors) [9].

The most common causes of injuries in young adult and adult patients were road traffic accidents, in particular involving motorcycles. Motor vehicle collisions and falls remained the second and third cases of trauma, alternating over the years. The comparison with other studies on patterns of trauma was limited by the differences in population samples, methods of data collection, number of hospitals involved in the analyses and timeframes taken into account. However, the review of recent literature [10–16] provided interesting elements of analysis. The incidence of injuries following road traffic collisions has been increasing globally in the last two decades [10], with the exception of countries with high socio-demographic index (i.e. income per capita, average educational attainment and total fertility rate). However, in European countries, non-fatal road-related injuries still pose a relevant burden to the society [11]. Dhondt et al.[12] highlight the health risk per kilometer travelled which they observed to be higher for vulnerable road users, i.e. those unprotected by outside shield, especially motorcyclists, cyclists and pedestrians.

In contrast with recent literature [13, 14] that reports a progressive increase in falls in Europe and USA together with the decrease in road traffic-related injuries, no significant rise in total number of falls was observed over the 9 years of this study. Concomitantly, the predominance of road traffic collisions did not change over the years. While young and adult people were more often victims of motorcycle and motor vehicle incidents, in extremes of age falls, bicycle and pedestrian accidents were predominant.

With the progressive ageing of the population, a significant increase in the age of trauma patients was expected, as observed in international literature [13, 15]. Interestingly, over the years of this study, the median age was constant with minimal oscillations around 38 years. This could be due to a preferential centralization to the TC of younger patients with complex multi-district trauma, while the elderly are often treated in level 2 TCs of the urban area.

Different from older patients, children were less likely to suffer severe lesions for the same mechanisms: it was observed that the ratio of the severely injured increased with age, from less than 1/6 of children to more than half of the elderly. For this reason, it was suggested to consider older age itself as a criterion of severity [17]. This conclusion is also supported by our results which demonstrated that age was an independent predictor of death. Even if the elderly patients were more likely to sustain severe lesions, the extremes of age always represented a smaller fraction of the total: most patients had an age comprised between 14 and 39 and, if considering the severely injured, between 40 and 64.

The age group of young adults (14–39 years old) includes the years of adolescence, between 14 and 20 years of age [18]. Adolescence is characterized by “reduced self-control, increased risk-taking behaviors and experimentation with alcohol and drugs” [16]. Roberts et al.[16] observed evidence of alcohol and drugs abuse in 20% adolescent trauma patients. Mental illnesses often present in adolescence and may lead to suicidal behaviors and Roberts et al. observed that adolescents with pre-existing psychiatric diagnosis suffer injuries with higher ISS [16]. Coherently, self-inflicted injuries became particularly relevant among severely injured young adults, overcoming accidental trauma and injuries caused by assault, the last of which was more frequently the cause of milder injuries in this age group. Further analysis of pre-existing psychiatric conditions may help the implementation of targeted interventions in patients at risk and the planning of adequate preventive measures, especially among the younger population.

This study has allowed to observe a clustering by gender in specific years of age, especially when considering the severely injured. Female patients showed a peculiar trimodal distribution: the three peaks correspond to a proportional increase in self-inflicted injuries around 20 years, mixed increase in self-inflicted and accidental injuries around 45 and a marked increase in accidental injuries at about 80 years of age. Male patients were generally injured in the central years of life, mostly in relation to road traffic accidents. Almost 90% of injured motorcyclists were male. Pileggi et al. [19] in their assessment of risk behaviors among adolescent motorcyclists reported that “males have a high preponderance of violations” which poses them at higher risk of being involved in motorcycle collisions than the female counterpart. Work-related injuries affected mostly men and were rarely found among women.

Logistic regression models showed no correlation of mortality with gender. MOI, age range and ISS ≥ 16 were found to be independent predictors of mortality. While the elderly were less affected by trauma than the younger part of the population, injuries were more likely to be deadly in older patients, as seen also in international literature [20, 21]. The crude number of the deceased increased with age and, different from what was reported by Rhee et al. [22], no relevant peaks were observed in younger age groups. This is consistent with the fact that in the twentieth century, great efforts and funding have been destinated to the fights against the two leading causes of death (cardiovascular diseases and cancer) [23], ameliorating the life expectancy. The improved health status of older people in our society explains the increased mobility and activity of this age group: the elderly are nowadays exposed to a similar risk of injury as younger people and the distribution of MOIs mirrors the progressive evolution in the definition of “who is old” in our society. Therefore, particular attention should be given to the prevention of trauma, as it constitutes the third cause of death, with focus not only on road traffic accidents but also on domestic incidents, especially falls.

Interpersonal violence is the least lethal type of trauma among the ones analyzed, probably due to the low number of victims of assault with firearms recorded in the trauma registry and the reduced lethality of the other means used, e.g. knives, battery, blunt objects. In contrast with the USA, in Europe, GSW are mostly considered a secondary concern [24]. Finland is one of the European countries with the highest firearm-related mortality; however, it was reported that almost half of GSW are unintentional, mostly related to diffusion of recreational hunting [25].

The main limitation of this study is that it is a single-center study. Despite the elevated number of trauma patients treated in Niguarda TC, further studies in conjunction with the newly created regional trauma registry may provide a more complete and population-based picture of the epidemiology of trauma and a useful insight on the functioning of the regional trauma system. The systematic introduction of trauma registries in Italy could contribute to decrease trauma morbidity and mortality [7, 31], through the improvement of different aspects (e.g. road safety, psychological support network for suicide prevention, pre-hospital care, etc.). To provide an epidemiological decryption of trauma distribution could be important to trauma prevention and management.

## Conclusion

In conclusion, this study allows the following observations:

- Males were more affected by severe trauma than females and blunt trauma was prevailing.
- Overtriage increased progressively, due to a low specificity of pre-hospital triage especially with regard to the use of high-energy mechanism as a criterion for centralization.
- Motorcycle and motor vehicle collisions are the main MOIs in young adults and adults, while pedestrian accidents and falls were more frequent in extremes of age.
- The relevance of severe and non-severe motorcycle-related injuries among male young adults and adults has not changed in the last 9 years. Further studies on the compliance to street regulations, and eventually the implementation of additional restrictive measures or the adjustment of the existing ones might be required.
- Severe injuries were clustered in middle age in males, while females showed a three peaks distribution during adolescence, middle age and older age.
- Overall mortality was low but increased with age. GSW were the most lethal followed by falls and bicycle accidents. Self-inflicted intentional injuries were more associated to the demise of the patients than non-intentional injuries and affected mostly adolescents and young adults. Early detection of psychiatric conditions and access to proper medical and psychological support for the youngest patients, both male and female, together with thorough follow-up of known patients, may prove useful in lowering the rates of self-harm with suicidal intent.

Finally, trauma remains a pathology of the young, but incidents, both domestic and related to road traffic, involving the geriatric population are increasingly becoming a concern in our ageing population. Effective preventive measures should be studied and applied to tackle the most relevant risks to reduce the burden of injuries on our society.

## Data Availability

The authors are responsible of the data described in the manuscript and assure full availability of the study material.

## References

[1] Krug EG, Sharma GK, Lozano R (2000). The global burden of injuries. Am J Public Health.

[2] Moore L, Clark DE (2008). The value of trauma registries. Injury.

[3] Dick WF, Baskett PJF (1999). Recommendations for uniform reporting of data following major trauma—the Utstein style: a report of a working party of the International Trauma Anaesthesia and Critical Care Society (ITACCS). Resuscitation.

[4] Ufficio Servizi statistici della Città metropolitana di Milano (2018). Dati statistico demografici dell’area metropolitana di Milano. http://www.cittametropolitana.milano.it/export/sites/default/statistica/doc/Statistiche_demografiche_al_1_gennaio_2018.pdf

[5] Ringdal KG, Coats TJ, Lefering R, Di Bartolomeo S, Steen PA, Røise O, Utstein TCD (2008). The Utstein template for uniform reporting of data following major trauma: a joint revision by SCANTEM, TARN, DGU-TR and RITG. Scand J Trauma ResuscEmerg Med.

[6] Orimo H, Ito H, Suzuki T, Araki A, Hosoi T, Sawabe M (2006). Reviewing the definition of “elderly”. Geriatrics GerontolInt.

[7] Chiara O (2008). The model of the Niguarda Hospital trauma team in Milan. Chir Ital.

[8] Uleberg O, Vinjevoll OP, Eriksson U, Aadahl P, Skogvoll E (2007). Overtriage in trauma–what are the causes?. ActaAnaesthesiolScand.

[9] Centro Nazionale per l'Eccellenza Clinica, la Qualità e la Sicurezza delle Cure (CNEC) (2008). Raccomandazioni della Linea Guida sulla Gestione Integrata del Trauma Maggiore dalla scena dell’evento alla cura definitive. https://snlg.iss.it/wp-content/uploads/2020/06/LGTM_Racc1_4_def.pdf

[10] James SL, Lucchesi LR, Bisignano C, Castle CD, Dingels ZV, Fox JT, Sylte DO (2020). Morbidity and mortality from road injuries: results from the Global Burden of Disease Study 2017.

[11] Weijermars W, Bos N, Filtness A, Brown L, Bauer R, Dupont E, Thomas P (2018). Burden of injury of serious road injuries in six EU countries. Accid Anal Prev.

[12] Dhondt S, Macharis C, Terryn N, Van Malderen F, Putman K (2013). Health burden of road traffic accidents, an analysis of clinical data on disability and mortality exposure rates in Flanders and Brussels. Accident Accid Anal Prev.

[13] Kehoe A, Smith JE, Edwards A, Yates D, Lecky F (2015). The changing face of major trauma in the UK. Emerg Med J.

[14] Khorgami Z, Fleischer WJ, Chen YJA, Mushtaq N, Charles MS, Howard CA (2018). Ten-year trends in traumatic injury mechanisms and outcomes: a trauma registry analysis. Am J Surg.

[15] Nijboer JM, van der Sluis CK, van der Naalt J, Nijsten MW, ten Duis HJ (2007). Two cohorts of severely injured trauma patients, nearly two decades apart: unchanged mortality but improved quality of life despite higher age. J Trauma Inj Infect Crit Care.

[16] Roberts Z, Collins JA, James D, Bouamra O, Young M, Lyttle MD, Mullen S (2020). Epidemiology of adolescent trauma in England: a review of TARN data 2008–2017. Emerg Med J.

[17] Demetriades D, Sava J, Alo K, Newton E, Velmahos GC, Murray JA, Berne TV (2001). Old age as a criterion for trauma team activation. J Trauma Acute Care Surg.

[18] Sawyer SM, Azzopardi PS, Wickremarathne D, Patton GC (2018). The age of adolescence. Lancet Child Adolesc Health.

[19] Pileggi C, Bianco A, Nobile CG, Angelillo IF (2006). Risky behaviors among motorcycling adolescents in Italy. J Pediatr.

[20] Perdue PW, Watts DD, Kaufmann CR, Trask AL (1998). Differences in mortality between elderly and younger adult trauma patients: geriatric status increases risk of delayed death. J Trauma Acute Care Surg.

[21] Clement ND, Tennant C, Muwanga C (2010). Polytrauma in the elderly: predictors of the cause and time of death. Scandinavian J Trauma.

[22] Rhee P, Joseph B, Pandit V, Aziz H, Vercruysse G, Kulvatunyou N, Friese RS (2014). Increasing trauma deaths in the United States. Ann Surg.

[23] Centers for Disease Control and Prevention (CDC) (1999). Decline in deaths from heart disease and stroke–United States, 1900–1999. MMWR Morb Mortal Wkly Rep.

[24] Hink AB, Bonne S, Levy M, Kuhls DA, Allee L, Burke PA, Stewart RM (2019). Firearm injury research and epidemiology: A review of the data, their limitations, and how trauma centers can improve firearm injury research. J Trauma Acute Care Surg.

[25] Mattila VM, Mäkitie I, Pihlajamäki H (2006). Trends in hospitalization for firearm-related injury in Finland from 1990 to 2003. J Trauma Acute Care Surg.

[26] Di Bartolomeo S, Sanson G, Michelutto V (2004). Epidemiology of major injury in the population of Friuli Venezia Giulia—Italy. Injury.

[27] Mamo C, Coggiola N, Dalmasso M (2019). Suicide epidemiology in Italy: a population-based study in Piedmont region. Eur J Public Health.

[28] Bianchi FP, Veneziani V, Cantalice MA (2018). Epidemiology of injuries among Italian footballers: the role of the playing field. InjPrev.

[29] Granieri SS, Reitano EE, Bindi FF (2020). Motorcycle-related trauma:effects of age and site of injuries on mortality. A single-center retrospective study. World J EmergSurg.

[30] Casati A, Granieri S, Cimbanassi S (2020). Falls from height analysis of predictors of death in a single-center retrospective study. J Clin Med.

[31] Gruen RL, Jurkovich GJ, McIntyre LK, et al. (2006) Patterns of errors contributing to trauma mortality: Lessons learned from 2594 deaths. Ann Surg 10.1097/01.sla.0000234655.83517.56 PMC1856538 16926563

